# Interaction between periodontal disease and colorectal cancer: implications of oral microbiota in carcinogenesis

**DOI:** 10.17179/excli2024-7869

**Published:** 2024-12-13

**Authors:** Thalles Yurgen Balduino, André Felipe dos Santos Teles, Gabriel Leonardo Magrin, Marco Aurélio Bianchini

**Affiliations:** 1 Department of Dentistry, Center for Education and Research on Dental Implants (CEPID), Federal University of Santa Catarina (UFSC), Florianópolis, Brazil

## ⁯

Colorectal cancer (CRC) is the third most diagnosed cancer globally and the second leading cause of cancer-related deaths worldwide. In 2020, there were over 1.9 million new cases and approximately 900,000 deaths, according to data from the Global Cancer Observatory of the World Health Organization (Idrissi Janati et al., 2022[3]; Sung et al., 2021[10]). Even though CRC can present higher occurrence rates in individuals with inflammatory bowel diseases or hereditary syndromes, most cases occur sporadically, meaning there is no clearly defined genetic cause (Ponz de Leon and Percesepe, 2000[8]).

Multiple factors can contribute to the development of sporadic colorectal cancer. Among them are advanced age, diabetes mellitus, obesity, family history of CRC, smoking, and excessive alcohol consumption (Idrissi Janati et al., 2022[3]). In recent years, research has indicated that periodontal disease (PD), a chronic inflammatory condition of the gums, may also be associated with an increased risk of developing colorectal cancer (Idrissi Janati et al., 2022[3]; Madugula et al., 2024[4]).

Periodontal disease is a chronic inflammatory condition caused by an imbalance in the bacterial biofilm that forms on teeth's surface (Papapanou et al., 2018[6]). Under normal conditions, there is a balance between beneficial and pathogenic bacteria and the host's immune system. However, the accumulation of biofilm beyond the host's defense capacity can lead to gums inflammation (gingivitis). If not addressed, this inflammation can advance to periodontitis, a more severe stage of the disease.

Periodontitis can be associated with other medical conditions beyond the mouth, such as cardiovascular diseases, respiratory diseases, diabetes, complications during pregnancy, and even cancer. This occurs because periodontal pathogens and their toxins can spread to other parts of the body through the bloodstream, causing systemic inflammation (Nazir, 2017[5]). Chronic inflammation has been widely recognized as an important risk factor for carcinogenesis, contributing to genetic mutations, inhibition of apoptosis (programmed cell death), stimulation of angiogenesis (formation of new blood vessels), and uncontrolled cell proliferation (Sears and Queen, 2024[9]).

The mechanism that connects periodontitis to colorectal cancer involves the dissemination of periodontal bacteria, their inflammatory products, and endotoxins to the intestine, creating a favorable environment for chronic inflammation and tumor promotion (Supplementary Figure 1). This association between periodontal disease and gastrointestinal cancers has been investigated, although the strength of the evidence varies among different types of cancers (Castellarin et al., 2012[1]).

*Fusobacterium nucleatum* (Fn), a periodontal pathogen from the orange complex present in the oral cavity has been widely associated with colorectal cancer (CRC), although its role in disease progression is not yet fully understood (Idrissi Janati et al., 2022[3]; Madugula et al., 2024[4]). This bacterium may influence carcinogenesis through mechanisms such as immune evasion, where it inhibits the adaptive immune response, and via its adhesion proteins (FadA and Fap2), which facilitate the invasion of tumor cells (Pignatelli et al., 2023[7]). Additionally, *F. nucleatum* induces chronic inflammation, which promotes mutations and the malignant cells proliferation, and modulates the tumor microenvironment, creating conditions that stimulate tumor growth and spread (Pignatelli et al., 2023[7]). Studies indicate that periodontal disease (PD) may increase the risk of CRC by promoting systemic inflammation, a potential carcinogenic factor in distant organs like the colon (Idrissi Janati et al., 2022[3]; Madugula et al., 2024[4]). Thus, the link between poor oral health and gastrointestinal cancer suggests that PD may be an important modifiable risk factor for CRC prevention.

Individuals with a history of periodontal disease (PD) exhibited a 1.45 times higher risk of developing colorectal cancer (CRC), indicating a significant correlation between the severity of PD and the likelihood of CRC (Idrissi Janati et al., 2022[3]). 

The identification of periodontal disease (PD) as a modifiable risk factor for colorectal cancer (CRC) reinforces the importance of oral health in cancer prevention. Therefore, interventions aimed at treating and preventing PD could help reduce the risk of CRC and possibly reduce its incidence (Idrissi Janati et al., 2022[3]).

Although the translocation of pathogenic bacteria from the oral cavity to the intestine has been minimal, the presence of these bacteria in the tumor environment, especially *Fusobacterium nucleatum* is correlated with poorer prognosis and resistance to chemotherapy (Idrissi Janati et al., 2022[3]; Pignatelli et al., 2023[7]). Thus, therapeutic strategies focused on blocking the colonization or interactions between *F. nucleatum* and tumor cells, such as the use of probiotics or antibiotics, emerge as promising approaches (Sears and Queen, 2024[9]). Additionally, the genotypic and phenotypic variation of *F. nucleatum* points to this bacterium's capacity to adapt, influenced by gene recombination and horizontal transfer, suggesting that modulating the microbiome could be a viable strategy to prevent or treat colorectal cancer (Crowley et al., 2024[2]).

The conclusion of this body of studies reinforces the essential role of the microbiota, particularly *Fusobacterium nucleatum*, in the progression of colorectal cancer (CRC) and highlights oral health as a significant systemic risk factor. Furthermore, the strong correlation between periodontal disease (PD) and the increased risk of CRC underscores the need for integrated preventive strategies, where managing oral health plays a crucial role in reducing the risks of serious systemic diseases. These findings encourage ongoing research into the connections between oral health, systemic inflammation, and carcinogenesis, promoting a broader approach to CRC prevention with positive public health impacts.

The conclusion of this set of studies reinforces the essential role of the microbiota, particularly *Fusobacterium nucleatum*, in the progression of colorectal cancer (CRC) and highlights oral health as a relevant systemic risk factor. Furthermore, the strong correlation between periodontal disease (PD) and the increased risk of CRC points to the importance of integrated preventive strategies, since oral health management plays a crucial role in reducing the risks of serious systemic diseases.

These findings encourage the continuous research, investigating the connections between oral health, systemic inflammation, and carcinogenesis, promoting a broader approach to the prevention of colorectal cancer (CRC), with positive impacts on public health.

## Conflict of interest

None to declare.

## Supplementary Material

Supplementary information
