# Supplementary material for: Interaction between periodontal disease and colorectal cancer: implications of oral microbiota in carcinogenesis
Source: EXCLI J. 2024 Dec 13;23:1493–5. doi: 10.17179/excli2024-7869 (PMC11713988; doi:10.17179/excli2024-7869)
Supplement: Supplementary information [file EXCLI-23-1493-s-001.pdf]

EXCLI Journal 2024;23:1493-1495 – ISSN 1611-2156
Received: September 23, 2024, accepted: December 02, 2024, published: December 13, 2024

Letter to the editor:

INTERACTION BETWEEN PERIODONTAL DISEASE AND COLORECTAL CANCER: IMPLICATIONS OF ORAL MICROBIOTA IN CARCINOGENESIS

Thalles Yurgen Balduino*, André Felipe dos Santos Teles, Gabriel Leonardo Magrin, Marco Aurélio Bianchini

Department of Dentistry, Center for Education and Research on Dental Implants (CEPID), Federal University of Santa Catarina (UFSC), Florianópolis, Brazil

* **Corresponding author:** Thalles Yurgen Balduino, Department of Dentistry, Center for Education and Research on Dental Implants (CEPID), Federal University of Santa Catarina (UFSC), 88040-900 Florianópolis, Santa Catarina, Brazil.
E-mail: thallesbalduino@hotmail.com

https://dx.doi.org/10.17179/excli2024-7869

This is an Open Access article distributed under the terms of the Creative Commons Attribution License (http://creativecommons.org/licenses/by/4.0/).

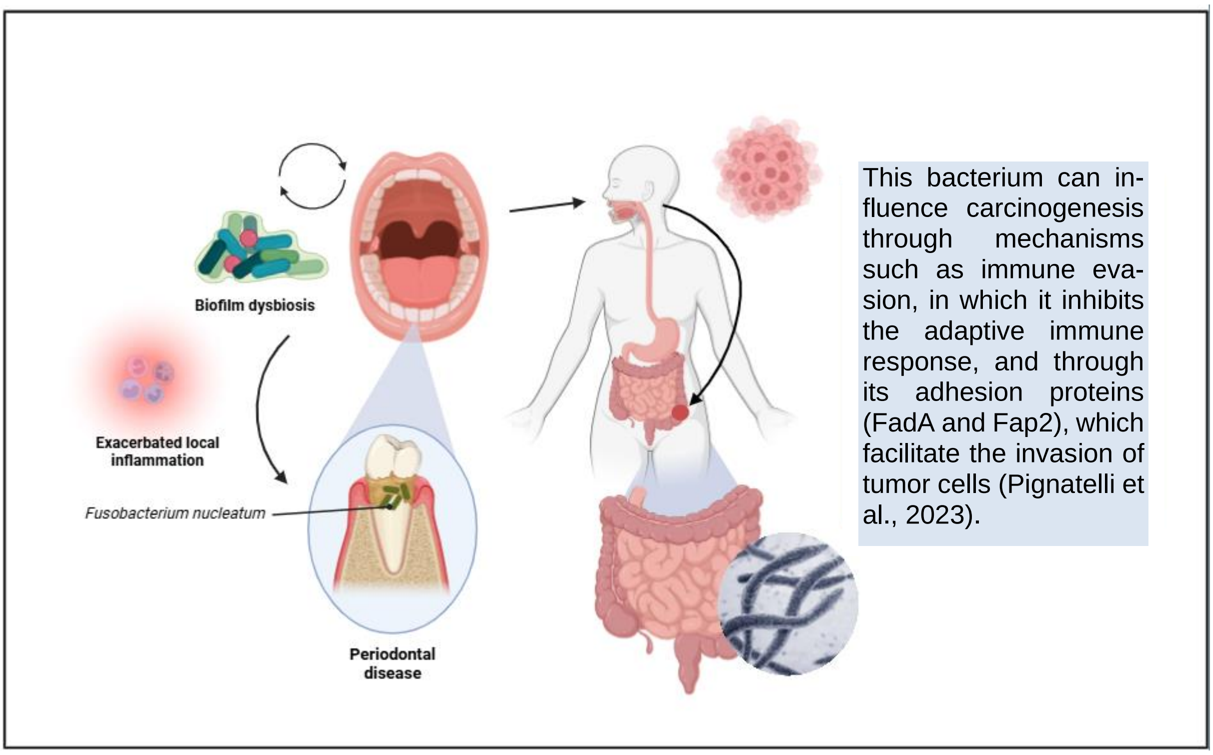

Supplementary Figure 1: *Fusobacterium nucleatum* role in the connection between periodontal disease and colorectal carcinogenesis.

Note: The oral dysbiosis and exacerbated local inflammation may facilitate the pathogens' translocation from the oral cavity to the gastrointestinal tract, contributing to the growth and development of colorectal tumors.
